# OA16 A case of anti-MDA5 dermatomyositis: the challenge of atypical skin manifestations

**DOI:** 10.1093/rap/rkad070.016

**Published:** 2023-09-27

**Authors:** Kathryn Biddle, Olivia Buckeldee, Israa Al-Shakarchi

**Affiliations:** Kingston Hospital, London, United Kingdom; Kingston Hospital, London, United Kingdom; Kingston Hospital, London, United Kingdom

## Abstract

**Introduction:**

Anti-MDA5 dermatomyositis (DM) is a rare systemic autoimmune condition associated with clinically amyopathic DM and rapidly progressive interstitial lung disease (RP-ILD). In addition to the classical DM dermatological manifestations, anti-MDA5 DM is associated with unique skin features including cutaneous ulceration, eyelid oedema, palmar papules and mechanic’s hands. An awareness of these features is essential for prompt diagnosis and management. Here we present a challenging case of anti-MDA5 DM with unusual skin manifestations, complicated by RP-ILD.

**Case description:**

A 60-year-old Chinese lady with no past medical history presented to ophthalmology with oedema to her left eye and was diagnosed with pre-septal cellulitis. One week later, she presented with low-grade fevers, fatigue, and palpitations on exertion. Systems review and blood tests were unremarkable, and she was discharged with outpatient cardiac follow-up.

One month later, she presented to the A&E with ongoing fatigue, 10kg unintentional weight loss, and reduced mobility. She was cachectic with ulcerating skin lesions on her shoulders and hips (thought to be pressure sores). Initial results showed a normocytic anaemia, deranged electrolytes (likely related to poor oral intake) and a raised troponin (likely rate-related). A CT scan of her chest, abdomen and pelvis showed no evidence of malignancy, but demonstrated patches of consolidation in both lungs, with nodularity and ground-glass changes; a differential diagnosis included inflammation, infection and heart failure. She developed pyrexia and mild hypoxia during admission, and was empirically treated for bilateral pneumonia.

Five days after admission, fasciculations in her upper limbs and tongue prompted a neurology review. Nerve conduction studies demonstrated myopathic changes. An elevated creatine kinase (CK) and an MRI lower limbs confirming diffuse oedema, further supported a myositis diagnosis. Concurrently, she was noted to have mechanic’s hands and a lesion, potentially representing a Gottron’s papule, was biopsied revealing features suggestive of an immune vasculitis.

Fifteen days after admission, she developed severe type-1 respiratory failure requiring transfer to intensive care. Repeat CT imaging showed worsening bilateral consolidation and ground-glass changes, with moderate pleural effusions.

Her rapidly progressively respiratory failure in the context of likely DM prompted a suspicion of anti-MDA5 DM, and urgent referral to a tertiary centre for extra-corporeal membrane oxygenation (ECMO) and aggressive immunosuppression. Unfortunately, she died three weeks later. Autoantibody testing confirmed positive anti-MDA5 and Ro52 antibodies.

**Discussion:**

This case presented a number of diagnostic challenges. Firstly, the patient’s initial presentation was not typical for classical DM. On admission, she had normal power in her upper and lower limbs and a normal CK. Furthermore, her skin manifestations were atypical and misdiagnosed as more common conditions. The skin ulceration on her shoulders and hips was attributed to pressure sores, and her eyelid oedema was thought to be secondary to pre-septal cellulitis. Throughout her inpatient stay, more typical features of DM were noted. These included a Gottron’s papule, mechanics hands and a proximal myopathy. When these features were detected, a diagnosis of DM was suspected, and the relevant investigations performed.

The second diagnostic challenge related to the diagnosis of RP-ILD. When the patients respiratory symptoms deteriorated, she was initially managed for pneumonia and fluid overload. After dermatology, rheumatology and respiratory review, a diagnosis of anti-MDA5 RP-ILD was suspected, and appropriate management was initiated. Unfortunately, due to the aggressive nature of her underling lung disease, she continued to deteriorate and died. This case highlights the significant mortality associated with this condition, and the importance of prompt recognition for optimisation of treatment.

**Key learning points:**

The hallmark cutaneous features of DM occur with a similar prevalence in patients with anti-MDA-5 DM. These include Gottron’s papules, a heliotrope rash, the Shawl sign, V sign and mechanics hands. Anti-MDA5 DM is also associated with specific dermatological manifestations including deep and painful skin ulceration, palmar papules and eyelid oedema. An awareness of these cutaneous features is important to allow earlier consideration of the diagnosis of anti-MDA5 DM.

Anti-MDA5 DM is commonly associated with clinically amyopathic or hypomyopathic dermatomyositis. Cohort studies estimate that between 23% − 100% of patients with clinically amyopathic DM test positive for anti-MDA-5 antibodies. Therefore, there should be a high index of suspicion for anti-MDA5 DM in patients with amyopathic presentations.

Anti-MDA-5 dermatomyositis is associated with RP-ILD and a poor prognosis. Observational studies have estimated that ILD occurs in 82 − 100% of patients with anti-MDA-5 DM in East Asian populations. RP-ILD has been estimated to occur in 39-100% of these patients, and is associated with an extremely poor prognosis (Nombel et al, 2021).

Observational studies suggest that CRP, ferritin and MDA-5 antibody titres correlate with mortality rates and treatment response. Further work is needed to identify biomarkers predictive of the development of RP-ILD.

A multidisciplinary team approach is vital in order to optimise the management of these patients, who can present to a broad range of speciality teams.

